# Instant Upcycling of Microplastics into Graphene and Its Environmental Application

**DOI:** 10.1002/smsc.202400176

**Published:** 2024-08-07

**Authors:** Muhammad Adeel Zafar, Mohan V. Jacob

**Affiliations:** ^1^ Electronics Materials Lab College of Science and Engineering James Cook University Townsville QLD 4811 Australia

**Keywords:** graphene, microplastics, plasma, sustainable synthesis

## Abstract

Microplastic pollution poses a growing threat to ecosystems globally, necessitating sustainable solutions. This study explores upcycling microplastics into graphene as a promising approach Traditional methods like pyrolysis and catalytic carbonization are slow and compromise graphene quality. Flash Joule heating is fast but energy‐intensive and hard to control. In contrast, atmospheric pressure microwave plasma (APMP) synthesis, the proposed technique, offers a one‐step, environmentally friendly alternative. APMP operates at relatively lower temperatures, reducing energy consumption and providing precise control over process parameters. This study demonstrates that polyethylene microplastics from waste dropper bottles can be efficiently transformed into graphene using APMP synthesis. Raman spectroscopy of synthesized material reveals a spectrum characteristic of graphene‐based materials, with indications of defects and the presence of oxygen content. X‐ray diffraction illustrates the characteristic graphitic lattice, with a slightly larger interlayer spacing attributed to intercalated functional groups. X‐ray photoelectron spectroscopy confirms sp^2^ hybridized carbon as the major component. High‐resolution transmission electron microscopy provides insights into the multilayered structure and variations in interlayer spacing. The as‐synthesized pristine graphene exhibits nearly ten times greater efficiency in adsorbing perfluorooctanoic acid compared to the oxidized form of graphene, although it is slightly less effective than graphene‐based nanocomposites.

## Graphical abstract

This study demonstrates the upcycling of polyethylene microplastics into high‐quality graphene using atmospheric pressure microwave plasma (APMP) synthesis. APMP offers a sustainable, energy‐efficient alternative to traditional methods, producing graphene with enhanced properties for specific applications. The graphene shows significant potential for environmental remediation, effectively adsorbing perfluorooctanoic acid from water.

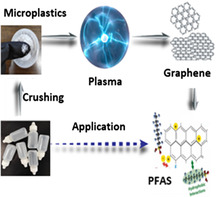

## 1 Introduction

Plastic pollution has surged to the forefront of concerns for both the scientific community and environmentalists. What compounds this issue is the degradation of plastic into smaller fragments, often reaching micron sizes, driven by a complex interplay of chemical, physical, and biological processes in the environment. These minuscule particles, known as “microplastics” typically consist of solid polymer particles measuring 1–5 mm.^[^ ^1^ ^]^ Microplastics are notorious for their nondegradable and insoluble nature in water, which is an evolving threat to aquatic and terrestrial ecosystems.^[^ ^2^ ^]^ For instance, the accumulation of microplastics in aquatic ecosystems ultimately integrates them into both marine and human food chains, while their high surface area‐to‐volume ratio enables them to absorb organic pollutants, further complicating the threat to living species.^[^ ^3^ ^]^ Microplastics originate from diverse sources, including raw materials used in pellets, textiles, and personal care products, and they pervade various environments, from soil and groundwater to plants, and oceans.^[^ ^4^, ^5^, ^6^, ^7^ ^]^ Polyethylene (PE), polypropylene (PP), polystyrene (PS), and polyethylene terephthalate (PET) are among the plastics most commonly identified as contributors to this escalating environmental issue.^[^ ^8^ ^]^ The global recognition of the eradication of microplastic pollution as a matter of utmost importance is now widespread.

Various technologies have been considered for the remediation of this issue, including recycling, degradation, and upcycling. The conventional approach of recycling faces significant challenges due to labor‐intensive separation processes and high costs, resulting in less than 10% of plastic waste being recycled in the United States in 2018.^[^ ^9^ ^]^ Upcycling, which involves transforming plastic waste into higher‐value materials rather than simply breaking it down into less valuable forms, is increasingly becoming a preferred approach. Various methods, such as pyrolysis,^[^ ^10^, ^11^, ^12^ ^]^ chemical vapor deposition (CVD),^[^ ^13^ ^]^ catalytic carbonization,^[^ ^14^ ^]^ and flash Joule heating (FJH)^[^ ^15^, ^16^, ^17^ ^]^ have been employed to transform waste plastics into graphene, an incredibly valuable and futuristic material. However, these methods come with drawbacks, including the requirement of specific substrates, catalysts, high vacuum conditions, etc.^[^ ^18^ ^]^ Moreover, these techniques are susceptible to potential contaminations from various sources, such as parent materials or during the transfer of graphene which can compromise the quality of graphene.^[^ ^19^ ^]^ **Table** **1** provides a comprehensive comparison of these techniques.

**Table 1:** Comparative analysis of microplastics and macroplastics upcycling techniques.

Plastic material	Method	Advantages	Disadvantages	References
Polystyrene obtained from Petri dishes (macrosize feedstock)	CVD	–Process feasibility for macrosize plastics –High *I*_2D_/*I*_G_ ratio and small *I*_D_/*I*_G_ ratio	–Substrate dependent –Requires high vacuum and preheating –Long process –Graphene transfer complications –Unscalable	[47]
Plastic bottles and packaging material (macrosize feedstock)	CVD	–Flexible and foldable paper‐like graphene foil –Various types of plastic materials were tried which showed good Raman characteristics –High *I*_2D_/*I*_G_ ratio and small *I*_D_/*I*_G_ ratio	–Substrate dependent –Requires high vacuum and preheating –Long process –Graphene transfer complications –Unscalable	[48]
PE and PS obtained from packaging material (macrosize feedstock)	Thermal decomposition + CVD	–Atmospheric pressure synthesis –Absence of D‐peak in Raman spectrum	–Two‐stage process –Substrate dependent –Requires preheating –Long process –Graphene transfer complications –Unscalable –Requires hydrogen gas	[13]
PET obtained from water bottles (macrosize feedstock)	CVD	–Waste gases from plastic pyrolysis are converted into graphene –Synthesis of monolayer graphene	–Requires copper film coating on a substrate –Long process –Graphene transfer complications –Unscalable	[49]
Polypropylene obtained from waste bumper and panel (macrosize feedstock)	Pyrolysis + carbonization	Scalable method	–Multistage process including pyrolysis, carbonization, purification –Use of toxic chemicals –Poor quality of graphene –20 layers graphene	[14]
Polypropylene ash obtained after its pyrolysis (macrosize feedstock)	Pyrolysis + FJH	Scalable technique	–Moderate *I*_2D_/*I*_G_ ratio and small *I*_D_/*I*_G_ ratio –High risk of doping and contamination due to no exhaust in the system –Postsynthesis grinding may contaminate and damage the graphene structure	[16]
Mixture of six polymers (microsize feedstock)	FJH	–High yield –Fast process –Do not require sorting of plastics	–Requires conductive material for synthesis such as carbon black –Prone to contamination –Moderate *I*_2D_/*I*_G_ ratio and small *I*_D_/*I*_G_ ratio	[15]
Mixture of polymers obtained from vehicle (microsize feedstock)	FJH	–High yield –Fast process –Do not require sorting of plastics	–Requires conductive material for synthesis such as coke –Prone to contamination –Moderate *I*_2D_/*I*_G_ ratio and small *I*_D_/*I*_G_ ratio	[17]
PE obtained by crushing dropper bottles (microsize feedstock)	APMP	–Substrate‐free synthesis at ambient conditions –Fast, single‐step process –Contamination‐free production	–Low yield of graphene –Moderate *I*_2D_/*I*_G_ ratio and small *I*_D_/*I*_G_ ratio	This work

A recently emerging technique known as atmospheric pressure microwave plasma (APMP) offers a significant advantage in graphene production. Using APMP, diverse precursors like methane, ethanol, and oil vapors have been successfully transformed into contaminant‐free, high‐quality graphene.^[^ ^20^, ^21^ ^]^ Notably, this process is conducted under ambient conditions, eliminating the necessity for high vacuum, substrates, or catalysts. However, its applicability is restricted to gaseous or vapor‐phase precursors, neglecting the potential of solid precursors.^[^ ^22^, ^23^, ^24^, ^25^, ^26^ ^]^

In this study, the goal is to broaden the application of APMP to solid precursors, specifically converting microplastics into graphene. In contrast to the traditional method of initiating graphene production from gaseous‐phase products, this approach involves the transformation of PE microplastics into gases such as methane, ethylene, and ethane, and then converting them into graphene within the plasma, all in one step. Furthermore, the advantages of microwave‐based technologies in terms of energy consumption and cost compared to conventional techniques for recycling or upcycling polymers can be found in recently reported studies.^[^ ^27^, ^28^, ^29^ ^]^ Following the successful synthesis of graphene, we also showcase its effectiveness in adsorbing perfluorooctanoic acid (PFOA), facilitated by ultrasonication.

## 2 Experimental Section

**Figure 1:**
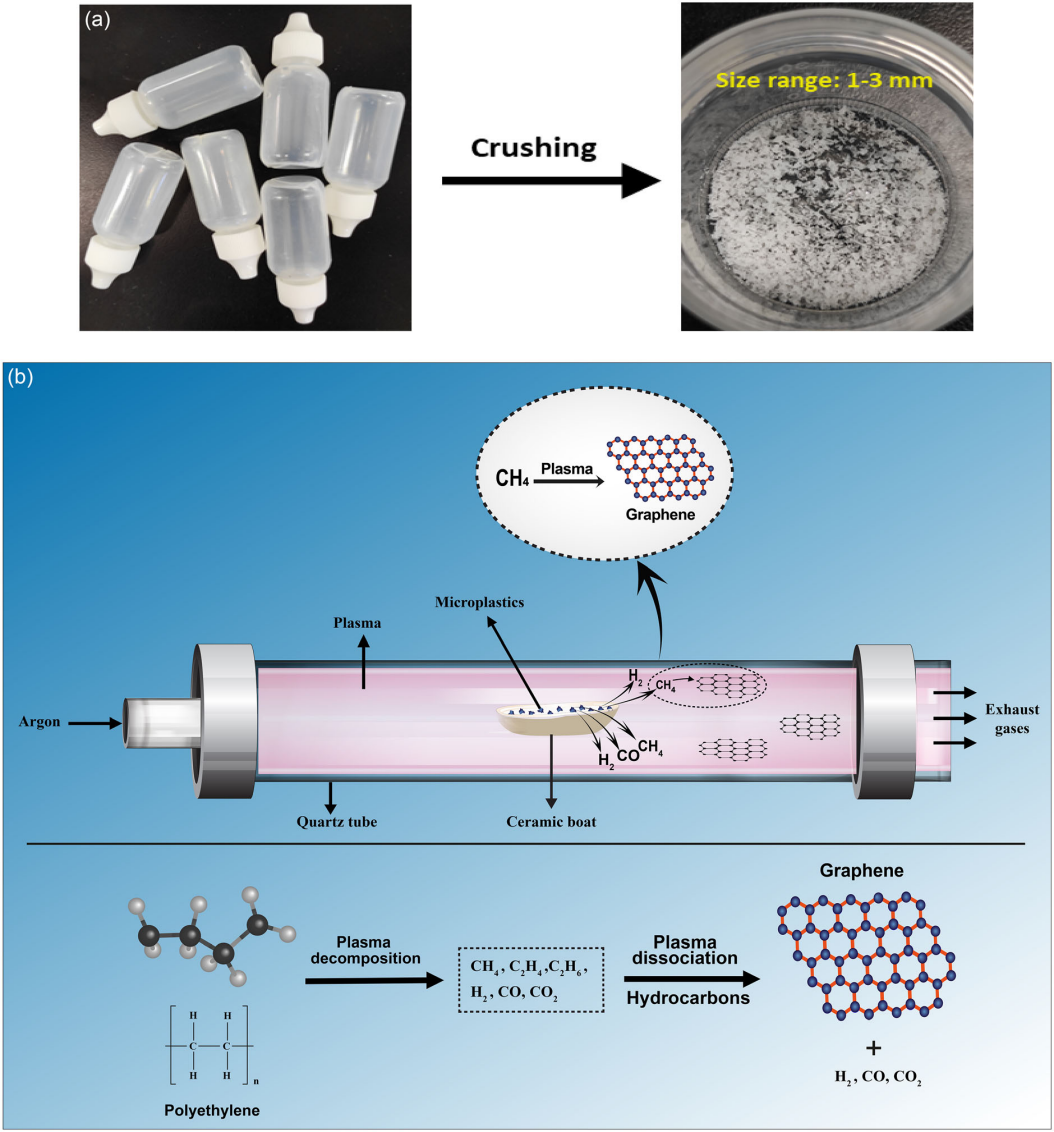
a) The figure depicts dropper bottles that were crushed (without caps) into microplastics (1–3 mm sizes). b) Schematic representation of APMP system for the synthesis of graphene from PE microplastics.

To begin, clean PE microplastics were obtained by crushing PE dropper bottles using a household blender. Fourier transform infrared (FTIR) spectra of the crushed microplastics (Figure S1, Supporting Information) confirm their PE bonding structure. These microplastics were then sieved to achieve uniform particle sizes ranging from 1 to 3 mm. Subsequently, 30 mg of the sieved microplastics were placed in a ceramic boat positioned at the center of a quartz tube within the plasma system. The plasma system consists of a 2.45 GHz microwave power supply, a tuner, and a quartz reaction tube (30 mm OD), with argon gas used as the background gas to provide an oxygen‐free atmosphere. The operating conditions include microwave power levels of 400, 500, and 600 W, with an argon gas flow rate of 2 slm and reaction time of 1 min. The optimal conditions mentioned were established through extensive parametric investigations, although detailed parameters are not provided here. The graphene nanosheets synthesized through this approach are free‐standing and can be collected at the open end of the tube and on silicon substrate, or they may accumulate on the walls of the quartz tube. **Figure** **1** schematically depicts the synthesis process.

The synthesis mechanism inside plasma can be divided into two stages. Initially, the plasma efficiently breaks down the microplastics, converting them into constituent gases such as methane (CH_4_), ethylene (C_2_H_4_), ethane (C_2_H_6_), carbon dioxide (CO_2_), hydrogen (H_2_), and carbon monoxide (CO). Subsequently, hydrocarbons, particularly methane undergo further processing within the system, experiencing plasma dissociation and conversion into graphene. This graphene is deposited onto the walls of the quartz tube, from which it can be collected for subsequent analysis and characterization. The conversion of PE into constituent gases can be supported by previous studies,^[^ ^30^, ^31^, ^32^ ^]^ where PE is converted into various gases upon severe thermal degradation, either by plasma or conventional heating. These gases serve as precursors for further transformations.

### 2.1 Characterization Techniques

To characterize the vibrational properties of graphene, Raman spectroscopy was employed (Witec, excitation‐beam wavelength: 532 nm). The morphology of the graphene samples was analyzed using a field‐emission scanning electron microscope (SEM, Hitachi SU 5000). Prior to SEM observation, the samples underwent sputter coating with platinum. X‐ray diffraction (XRD) analysis was conducted using a Bruker D8‐Advance X‐ray diffractometer equipped with Cu Kα radiation (*λ* = 0.154 nm). FTIR was performed using a Spectrum‐100 spectrometer from Perkin Elmer, USA. The surface element composition of the graphene was assessed using X‐ray photoelectron spectroscopy (XPS), which was performed on a Kratos Axis Ultra XPS instrument featuring an Al Kα X‐ray source. To delve into the microstructure of the graphene nanosheets, high‐resolution transmission electron microscopy (HRTEM, Hitachi HF 5000) operating at 200 kV was employed.

## 3 Results and Discussion

**Figure 2:**
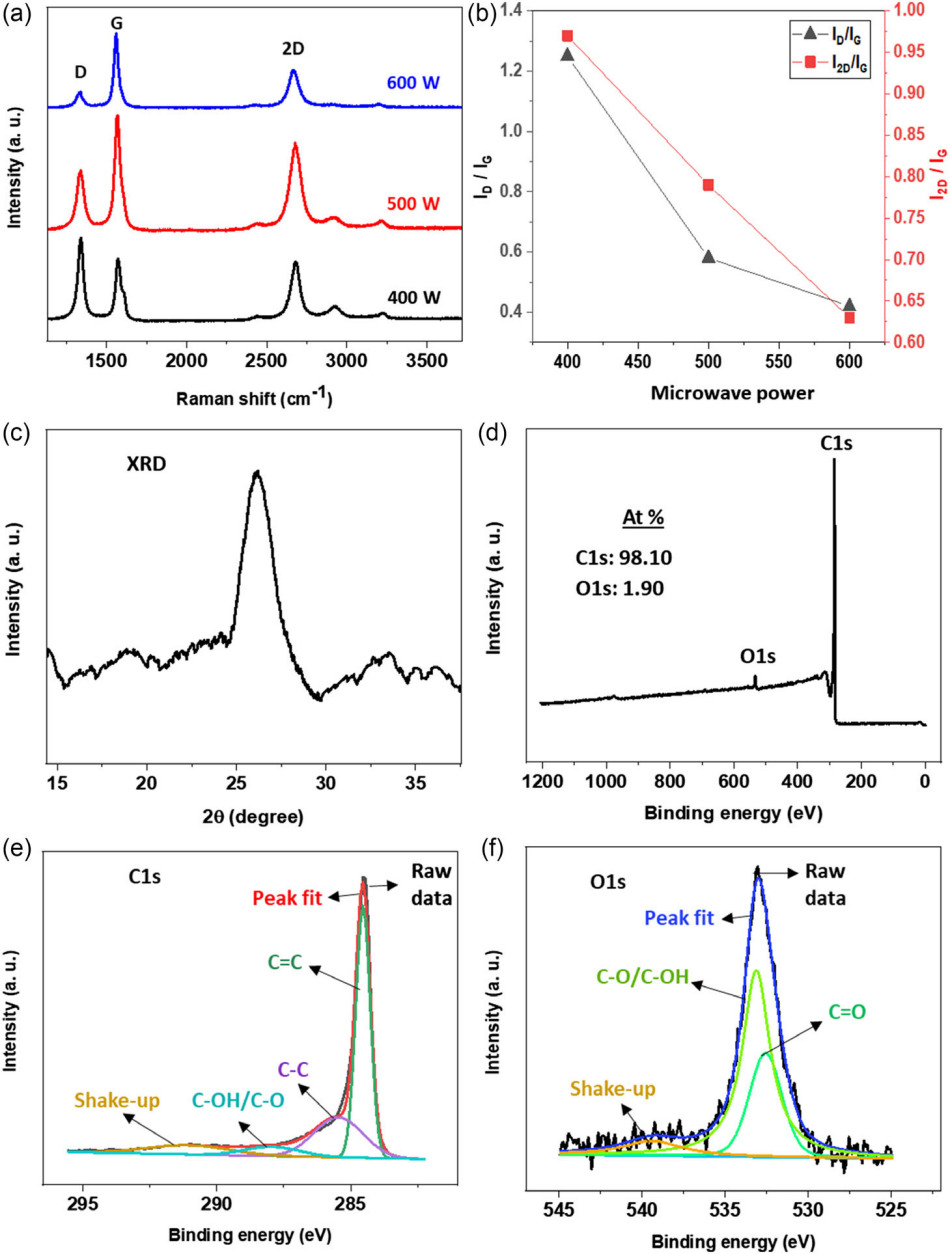
a) Raman spectra b) and the intensity ratios of the D and 2D peaks concerning the G peak in the graphene samples which demonstrate the influence of plasma power on the synthesis process. c) XRD pattern of 500 W graphene sample. 500 W graphene sample d) survey scan XPS, e) C1s high resolution, and f) O1s high resolution.

The as‐synthesized material was subjected to Raman spectroscopy for structural analysis. The Raman spectra of all samples (shown in **Figure** **2**a), regardless of the plasma power used for growth, exhibited three characteristic vibrational modes associated with graphene‐based materials: a D peak at ≈1333 cm^−1^ (indicative of defects), a G peak at ≈1576 cm^−1^ (related to vertical vibration), and a 2D peak at ≈2674 cm^−1^ (associated with two‐phonon vibration).^[^ ^33^ ^]^ The increase in temperature resulting from the rise in plasma power led to a reduction in the height of the D peak. This decline in intensity can be attributed to the removal of oxygen attached to graphene or the potential reduction in defects within the graphene structure. The intensity ratio of D and G peaks, i.e., *I*_D_/*I*_G_, which serves as a measure of disorder, is notably higher in the 400 W sample. This observation is consistent with prior research, such as the work of Marsden et al.^[^ ^34^ ^]^ where higher oxygen dosing in graphene exhibited a higher *I*_D_/*I*_G_ value. Conversely, the 500 and 600 W samples exhibited lower *I*_D_/*I*_G_ values, suggesting a reduced presence of defects or oxygen content compared to the 400 W sample. However, it can be noted that there were minimal differences in the *I*_D_/*I*_G_ or *I*_2D_/*I*_G_ ratios between the 500 and 600 W samples, as shown in Figure 2b. This indicates that microwave power beyond 500 W has a diminishing impact on the synthesis process.

The 2D peak in the Raman spectrum is a mark of graphene‐based materials, with characteristics like full width at half‐maximum (FWHM) and intensity ratio (*I*_2D_/*I*_G_) linked to the number of graphene layers. FWHM values of ≈30 cm^−1^ and *I*_2D_/*I*_G_ values of 2 or higher typically indicate monolayer graphene, while FWHM values of ≈50 cm^−1^ and *I*_2D_/*I*_G_ ratios of 1 to 1.5 suggest bilayer structures.^[^ ^21^ ^]^ The 500 and 600 W samples displayed similar FWHM values, measuring 84.8 and 78.3 cm^−1^, respectively, while the FWHM of the 400 W sample was 65.3 cm^−1^. Evaluating the *I*_2D_/*I*_G_ and FWHM values of samples, it can be inferred that all samples consist of multiple‐layer graphene. Nonetheless, the microwave power of 500 W is deemed suitable due to its lower *I*_D_/*I*_G_ value.

Crystalline materials exhibit an exclusive XRD pattern, often associated with a fingerprint for material identification. In Figure 2c, the characteristic peak of 500 W sample, positioned at a diffraction angle (2θ) of 26.3°, explicitly confirmed the effective creation of the graphitic lattice. This result aligns well with previous reports.^[^ ^35^, ^36^ ^]^ The peak position can be attributed to the (002) basal plane, denoting an interlayer spacing of 0.37 nm, a measurement further validated by TEM. It is noteworthy that this layer spacing slightly exceeds the 0.33 nm, usually reported for graphene. We ascribe this discrepancy to the presence of intercalated C—OH and C—O functional groups attached within the layers.

The 500 W graphene sample underwent XPS analysis to determine its elemental content and functional group types. The survey scan XPS (Figure 2d) revealed the presence of intense carbon (C) and small oxygen (O) peaks at 284.3 and 532.5 eV, respectively. The composition of C and O elements was ≈98.10% and 1.9%, respectively. High‐resolution spectra of C1s and O1s were further analyzed to study the bonding construction.

The C1s high‐resolution spectrum of 500 W sample was divided into four constituent peaks (Figure 2e). The primary C=C peak (sp^2^‐C), located at 284.3 eV, is vital for graphene material, and it indicates the occurrence of a honeycomb lattice structure. Additionally, the spectrum contained C—C (sp^3^‐C) and C—OH/C—O peaks at 285.2 and 288.1 eV, respectively. The sp^3^‐C peak was ascribed to either the edges of the graphene or doping effects within the structure.^[^ ^26^ ^]^ The O1s spectrum (Figure 2f) displayed impacts from O=C and C—OH/C—O peaks at 532 and 533 eV, respectively.^[^ ^37^, ^38^ ^]^ Comparing the XPS of 500 W sample with the 600 W sample (Figure S2, Supporting Information), it can be observed that the carbon content increased, whereas the oxygen content decreased slightly in the 600 W sample. Furthermore, in the C1s high‐resolution spectrum, sp^2^ bonding decreased and sp^3^ bonding increased compared to the 500 W sample. This could be related to the decreased 2D peak strength of the 600 W sample in the Raman spectrum. The differences in FWHM and peak positions observed in the XPS spectra of the 500 and 600 W samples can be attributed to surface charging effects, which can lead to shifts in binding energy positions, even though the samples were prepared on the same silicon substrate. Additionally, inhomogeneities within the samples, despite similar morphology in SEM images, may result in discrepancies in peak positions and FWHM. It should be noted that we used the same peak fitting conditions for all samples.

**Figure 3:**
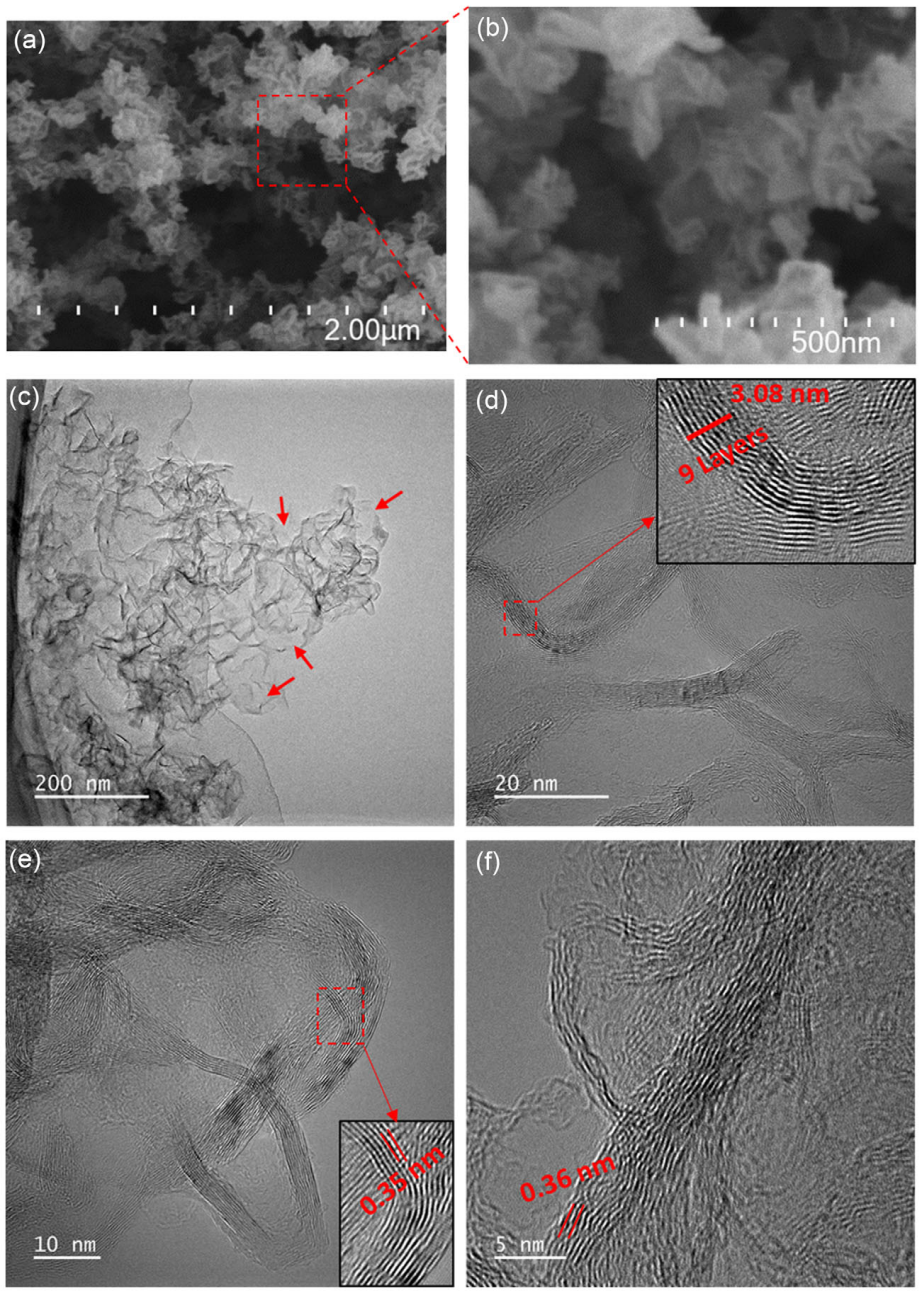
a,b) Low‐ and high‐magnification SEM images of 500 W graphene sample. c) Low‐magnification TEM and d–f) HRTEM images of 500 W graphene sample.

**Figure** **3**a,b displays low‐ and high‐resolution SEM images of the 500 W graphene nanosheets, directly deposited onto silicon substrate. As expected, the SEM images did not reveal smooth, two‐dimensional films. Instead, all the samples showcased typical three‐dimensional island‐like structures, resembling crumpled and folded sheets of paper scattered across the surface.^[^ ^39^ ^]^ This unique, curly, and wrinkled morphology predominantly stems from the inherent thermodynamic instability of two‐dimensional materials.^[^ ^40^ ^]^ Further validation of the primarily multilayered graphene composition comes from the HRTEM results, as depicted in Figure 3c–f. The high‐resolution SEM images of the 400 and 500 W samples in Figure S3, Supporting Information show a significant resemblance to the 500 W sample, with no apparent differences. The differences in Raman spectra despite the similar SEM images could be due to variations in the local chemical composition or structural defects that are not discernible in the SEM images. Raman spectroscopy is highly sensitive to molecular vibrations and can detect subtle changes in the material's structure and composition that are not visible in SEM. These variations could result from localized differences in the distribution of functional groups (oxygen functional groups in our case), strain, or defects within the graphene sheets. Thus, while the overall morphology appears consistent, the Raman spectra reveal underlying heterogeneities in the material's structural properties.

Low‐resolution TEM and HRTEM analyses were conducted on the 500 W graphene sample. The specimens used for TEM studies were mechanically detached from the silicon substrates. The TEM images in Figure 3c–f reveal the presence of both large and small graphene nanosheets. These graphene nanosheets exhibit an interesting resemblance to rippled silk waves, with some regions appearing transparent. The most transparent and seemingly featureless areas, as indicated by arrows in Figure 3c, are likely monolayer graphene nanosheets. In Figure 3d–f, high‐magnification TEM images provide further insights. The HRTEM images depicted that the graphene material consisted of multiple layers, as evidenced by the nine‐layer structure shown in the inset of Figure 3d. The images also unveiled an interlayer spacing ranging from 0.34 to 0.36 nm, nearly matching the (0 0 2) plane spacing as calculated from XRD analysis. The observed variances may be attributed to the presence of a small quantity of oxygen atoms occupying interstitial sites, inducing structural discontinuities, and promoting dislocation formation within the graphene lattice. HRTEM also revealed that while some graphene particles exist independently, most graphene sheets are interconnected, forming a large, crumpled structure due to agglomeration. This phenomenon results in partial overlapping and coalescence of the graphene sheets. The quantity of graphene produced in this approach mainly relies on the quantity of microplastic feedstock. ≈30 mg of microplastics produced nearly 5 mg of graphene in 1 min. This production rate is remarkably higher than that achieved in previously used gas‐phase synthesis in APMP, where the feedstock was ethanol,^[^ ^24^ ^]^ methane,^[^ ^22^ ^]^ or oil vapors.^[^ ^41^ ^]^

### 3.1 Experimental Procedure for PFOA Adsorption Using Synthesized Graphene

The graphene synthesized at 500 W was employed to assess its adsorption capacity for PFOA. In a related study by Lath et al.^[^ ^42^ ^]^ graphene oxide (GO) was utilized for adsorption using an orbital shaker, resulting in an adsorption efficiency of only 3.3% in a 3–4 h procedure. In the current investigation, we focused on pristine graphene with the assistance of ultrasonication at varying durations. Three identical samples were prepared by combining 10 mg of PFOA with 1 mg of graphene in 1 liter of distilled water within separate containers. Ultrasonication was conducted for durations of 15 and 30 min at a pH of 7 and at room temperature, using a specialized 40 kHz ultrasonicator with a power output of 30 W. Additionally, a comparative sample with a 30 min magnetic stirring method was prepared. After both ultrasonication and magnetic stirring, the samples underwent filtration using a 0.45 μm filter to effectively remove graphene nanosheets. Subsequently, the filtered samples were sent to the laboratory for analysis.

### 3.2 Adsorption Capacity of Graphene for PFOA

The removal efficiency was calculated using Equation (1), where *C*_0_ and *C*_e_ are the initial and final concentrations, respectively.

(1) $$R\left(\%\right)=\frac{C_{0}-C_{e}}{C_{0}}\times 100$$

**Figure 4:**
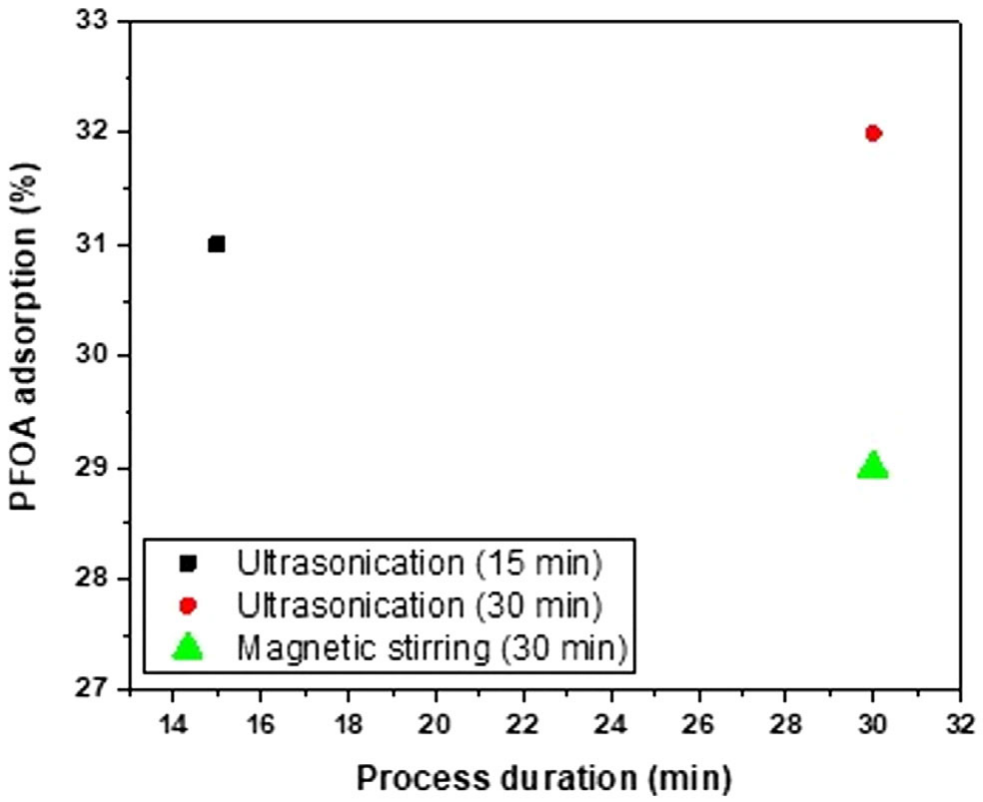
PFOA adsorption capacity on pristine graphene at different durations (15 and 30 min) with ultrasonication and magnetic stirring.

The graphical representation in **Figure** **4** represents the results of the adsorption study. The 15 min ultrasonicated sample exhibited a ≈30% adsorption capacity, which increased to ≈32% when the sonication time was extended to 30 min. Notably, when compared to the previously reported GO sample,^[^ ^42^ ^]^ which utilized an orbital shaker for enhanced adsorption and showed an efficiency of 3.3%, pristine graphene demonstrated significantly higher results. The increased adsorption capacity in this study is attributed to graphene's hydrophobic nature, fostering interactions with PFOA chains. This supports previous reports highlighting high adsorption capacities linked to the hydrophobic nature of carbon materials.^[^ ^43^, ^44^ ^]^ Ultrasonication likely contributed by increasing surface area and creating additional adsorption sites through improved dispersion and mass transfer. Ultrasonication surpassed magnetic stirring in combating agglomeration in graphene nanosheets. While both methods aid deagglomeration, ultrasonication's intensity ensures thorough exposure of nanosheets, maximizing accessible surface area for enhanced adsorption. However, it is essential to note that real‐world factors, including pH, temperature, and contaminants, can influence adsorption capacity.^[^ ^45^ ^]^ Furthermore, while the advantages of ultrasonication for adsorption are well known, a direct comparison of PFOA adsorption capacities between graphene synthesized in this study and GO using both ultrasonication and orbital shaking would provide a more comprehensive understanding of the influence of these agitation methods on adsorption performance.

Previously, graphene derivatives such as GO and fluorographene (FG), in composite with various other materials, have demonstrated excellent adsorption capacities exceeding 90%. However, pristine graphene, which possesses a higher adsorption capacity than GO, has not been investigated in this regard. For instance, GO composite with iron oxide^[^ ^42^ ^]^ showed more than 90% adsorption. Wang et al.^[^ ^46^ ^]^ synthesized a novel adsorbent by attaching hydrophobic FG to hydrophilic magnetic nanoparticles (MNPs). The resulting MNPs@FG exhibited impressive removal efficiencies of 91–97% for PFOA and perfluoro octane sulfonic acid. In an effort, to enhance this efficiency beyond 97%, it is proposed that incorporating pristine graphene with other materials may prove beneficial.

## 4 Conclusion

In summary, this research has successfully demonstrated the APMP synthesis as a rapid and facile method for fabricating graphene from microplastics, marking a significant milestone in this field. In comparison to established techniques such as CVD, pyrolysis, and FJH, the plasma‐based synthesis displayed superior characteristics, featuring contamination‐free production at ambient conditions. The method demonstrated the swift conversion of PE microplastics into graphene, a transformation confirmed by Raman spectroscopy, revealing graphene characteristics along with additional defects and oxygen content. XRD provided insight into the graphitic lattice, while XPS confirmed sp^2^ hybridized carbon. HRTEM offered a glimpse into the multilayered structure with interstitial spacing ranging between 0.34 and 0.36 nm. The pristine graphene synthesized through this process exhibited significant efficiency in adsorbing PFOA, positioning it as a promising candidate for addressing environmental challenges linked to microplastics. This research not only pioneers a novel approach to graphene synthesis but also contributes to the broader goal of mitigating the adverse effects of microplastic pollution on our ecosystems.

## Conflict of Interest

The authors declare no conflict of interest.

## Supporting information

Supplementary Material

## Data Availability

The data that support the findings of this study are available from the corresponding author upon reasonable request.
