# Supplementary material for: Instant Upcycling of Microplastics into Graphene and Its Environmental Application
Source: Small Sci. 2024 Aug 7;4(10):2400176. doi: 10.1002/smsc.202400176 (PMC11935150; doi:10.1002/smsc.202400176)
Supplement: Supplementary file 1 — Supplementary Material [file SMSC-4-2400176-s001.pdf]

<u>**Supplementary information**</u>

Instant upcycling of microplastics into graphene and its environmental application

*M. Adeel Zafar, Mohan V Jacob**

Electronics Materials Lab, College of Science and Engineering, James Cook University, Townsville, QLD 4811, Australia

**Email: mohan.jacob@jcu.edu.au*

Supplementary

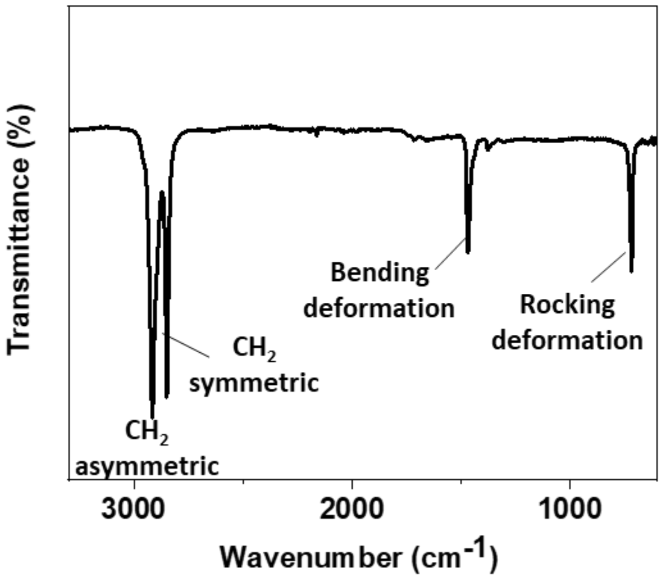

Figure S1: FTIR of crushed microplastics, confirming polyethylene characteristics

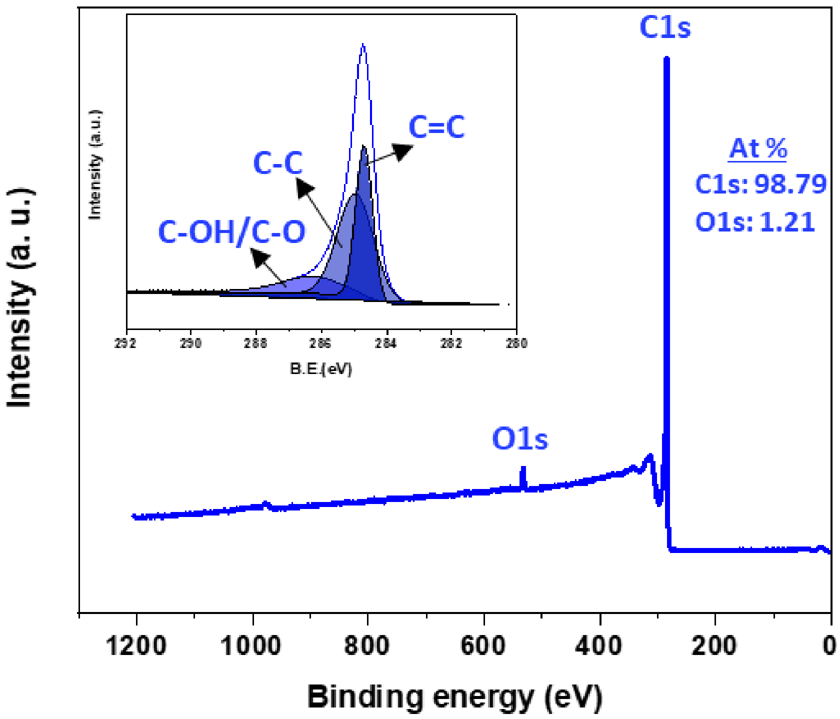

Figure S2: XPS survey and C1s scan of 600 W graphene sample

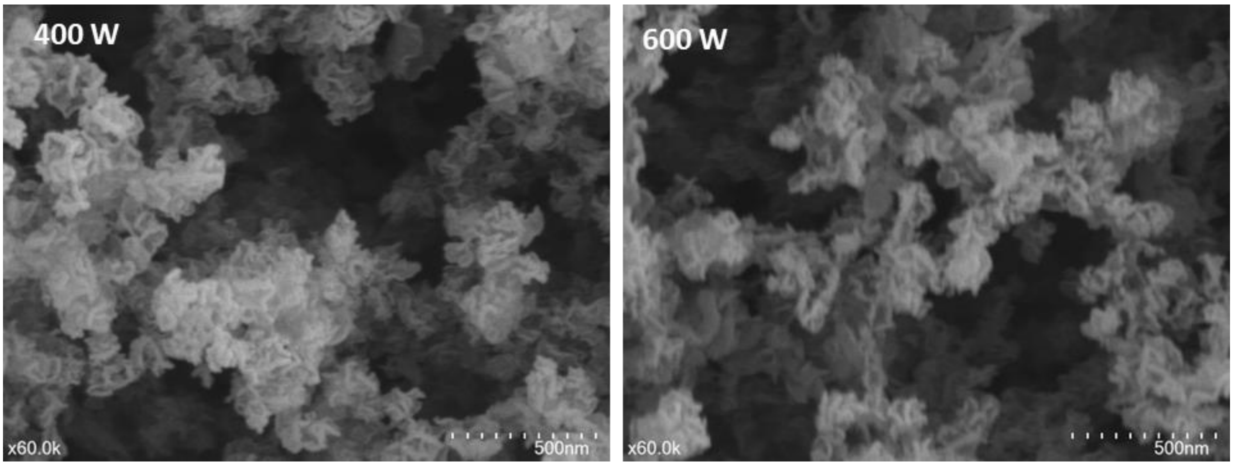

Figure S3: High-magnification SEM images of 400 and 600 W graphene samples
